# Negative valence of hallucinatory voices as predictor of cortical glutamatergic metabolite levels in schizophrenia patients

**DOI:** 10.1002/brb3.2446

**Published:** 2021-12-07

**Authors:** Helene Hjelmervik, Alexander R. Craven, Erik Johnsen, Kristiina Kompus, Josef J. Bless, Igne Sinkeviciute, Rune A. Kroken, Else‐Marie Løberg, Lars Ersland, Renate Grüner, Iris E. Sommer, Kenneth Hugdahl

**Affiliations:** ^1^ School of Health Sciences Kristiania University college Bergen Norway; ^2^ Department of Biological and Medical Psychology University of Bergen Bergen Norway; ^3^ NORMENT Center of Excellence Haukeland University Hospital Bergen Norway; ^4^ Division of Psychiatry Haukeland University Hospital Bergen Norway; ^5^ Department of Clinical Medicine (K1) University of Bergen Norway; ^6^ Department of Addiction Medicine Haukeland University Hospital Bergen Norway; ^7^ Department of Clinical Psychology University of Bergen Bergen Norway; ^8^ Department of Clinical Engineering Haukeland University Hospital Bergen Norway; ^9^ Department of Radiology Haukeland University Hospital Bergen Norway; ^10^ Department of Physics and Technology University of Bergen Bergen Norway; ^11^ Department of Neuroscience University Medical Center Groningen University of Groningen Groningen The Netherlands; ^12^ Department of Psychiatry University Medical Center Groningen University of Groningen Groningen The Netherlands

**Keywords:** auditory verbal hallucinations, BAVQ‐R, emotional valence, Glutamate, MR spectroscopy, schizophrenia

## Abstract

**Objectives:**

Negative emotional valence of auditory verbal hallucinations (AVHs) in schizophrenia can be a source of distress and is considered a strong predictor of illness severity. Previous studies have found glutamate to mediate AVH severity in frontal and temporal brain regions, however, they do not specifically address emotional valence of AVH. The role of glutamate for the experience of negative‐ versus positive emotional valence of AVH is therefore unknown and was investigated in the current study.

**Methods:**

Using magnetic resonance spectroscopy (MRS), 37 schizophrenia patients had Glx (glutamate+glutamine) measured in the left superior temporal gyrus (STG), and additionally in the anterior cingulate cortex (ACC) and the right STG, or in the left inferior frontal gyrus (IFG). Self‐reported emotional valence in AVH was measured with the Beliefs About Voices Questionnaire (BAVQ‐R).

**Results:**

Results from linear mixed models showed that negative emotional valence was associated with reduced Glx levels across all four measured brain regions in the frontal and temporal lobe. More specifically, voices that were experienced to be omnipotent (*p *= 0.04) and that the patients attempted to resist (*p* = 0.04) were related to lower Glx levels. Follow‐up analysis of the latter showed that voices that evoked emotional resistance (i.e., fear, sadness, anger), rather than behavioral resistance, was a significant predictor of reduced glutamate (*p* = 0.02).

**Conclusion:**

The findings could indicate aberrant glutamatergic signaling, or increased NMDA‐receptor hypoactivity in patients who experience their voices to be more emotionally negative. Overall, the study provides support for the glutamate hypothesis of schizophrenia.

## Graphical abstract

Negative emotional valence of auditory verbal hallucinations (AVHs) in schizophrenia can be a source of distress and is considered a strong predictor of illness severity. The current study investigated the molecular underpinnings of this symptom and found negative emotional valence in auditory hallucinations to be related to reduced Glx (glutamate+glutamine) in frontal and temporal brain regions. The results provide support for the glutamate hypothesis of schizophrenia.

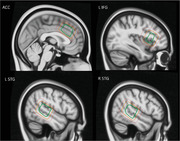

## 1 INTRODUCTION

Auditory verbal hallucinations (AVHs), auditory experiences in the absence of an external acoustic input (Ford et al., 2012; Hugdahl, 2009; Waters et al., 2006), are a key symptom in schizophrenia, and are present in about 70% of the patients (Mueser et al., 1990). This symptom can be a source of great distress, as hallucinations often take form of voices harassing or commanding the individual to do things against their will. However, the degree of negative emotional valence varies to a great extent between patients, and some patients even experience voices with positive and encouraging content (Cavelti et al., 2019). The degree of negative content of the voices has been shown to be a major factor to distinguish between clinical and nonclinical voice‐hearers (Daalman et al., 2011; Honig et al., 1998; see Laroi, 2012 for a review), and is a predictor of need for care (Honig et al., 1998).

The cognitive theory of AVHs (Chadwick & Birchwood, 1994) suggests that beliefs the patient has regarding the intent of the voice (malevolence or benevolence) and power of the voice (omnipotence) are critical for how the patient will handle and cope with the voice (van der Gaag et al., 2003). Further, the intent and power of the voice have been found to relate to two main types of emotional and behavioral responses. Patients who experience benevolent voices tend to engage with the voice by actively seeking and complying with intentions of the voice, while patients who report their voice to be of malevolent intent attempt to resist the voice through arguing and noncompliance (Chadwick & Birchwood, 1994). The beliefs about voice‐intent are often, but not always, related to the content of the voice. For example, a voice could be harsh, but the patient still believes it would look out for him/her (van der Gaag et al., 2003). Therefore, beliefs about voice content could arguably be even more tightly linked to distress than voice content in itself (Chadwick & Birchwood, 1994; Peters et al., 2011). The Beliefs About Voices Questionnaire (BAVQ (Chadwick & Birchwood, 1995), and the revised version, BAVQ‐R (Chadwick et al., 2000), are self‐report questionnaires that were developed to assess beliefs, feelings, and behavior related to AVH contents. The BAVQ‐R questionnaire consists of five subscales. Two subscales measure voice intent, the Malevolence and Benevolence subscales. Another subscale, the Omnipotence scale, captures how powerful the patient experiences the voice to be. In addition, the BAVQ‐R includes two additional sub‐scales, Resistance and Engagement, which capture the patient's relationship to the voice, that is, how the patient responds emotionally and behaviorally to the voice.

Malevolent voices are often associated with a resistive coping style, while benevolent voices are associated with an engaging coping style (Sayer et al., 2000). Based on factor analysis Strauss et al. (2018) suggested that malevolence and omnipotence are in essence overlapping, and hence suggested a two‐factor structure, called persecutory and benevolent beliefs—where the former can be argued to be characterized as unpleasant and of negative emotional valence, while the latter as more pleasant and of positive emotional valence. However, whether the degree of malevolence, omnipotence, and resistance on the one hand, and benevolence and engagement on the other hand, also relate to differences in the underlying neurobiology of AVHs is currently unknown.

Accumulating evidence suggests that glutamatergic dysfunction could play a role in the etiology of schizophrenia, and glutamate has in several studies been associated with symptom severity (see Merritt et al., 2013 for a review). A few studies have investigated glutamate and Glx (the sum of glutamate+glutamine) in relation to AVHs using magnetic resonance spectroscopy (^1^H‐MRS), an in vivo method that takes advantage of the magnetic properties of the hydrogen proton (de Graaf, 2007) to assess metabolite levels in brain tissue. Glx is often reported as glutamate and glutamine are difficult to separate accurately. It is usually glutamate that contribute the most to the Glx signal (Ramadan et al., 2013). Previous findings suggest that elevated Glx levels in the left superior temporal gyrus (STG) (Hjelmervik et al., 2020; Hugdahl et al., 2015), and inferior frontal gyrus (IFG) (Curcic‐Blake et al., 2017; Hugdahl et al., 2015) are associated with AVH severity (see also Jardri et al., 2016). Hjelmervik et al. (2020) also found a negative association for Glx and AVH severity in the ACC. These findings were interpreted as reflecting STG hyper‐ and ACC hypo‐activity, as has also been shown for fMRI (Hugdahl et al., 2009; Jardri et al., 2016). Previous studies relied on the Positive and Negative Syndrome Scale (PANSS) P3 item to assess AVH severity (e.g., Hjelmervik et al., 2020; Hugdahl et al., 2015). However, a limitation is that the PANSS scale does not allow for data on voice intent, emotional and behavioral coping strategies, with the result that these factors have not previously been investigated in relation to glutamate and Glx. Having established that Glx could mediate AVH, a natural next step in this research is therefore to investigate more specific aspects of AVH, such as emotional valence. Since, fMRI‐studies of emotional content of real voices suggest overlapping brain regions with those for auditory hallucinations, including temporal (STG) and frontal (ACC and IFG) regions (Bestelmeyer et al., 2017), one could hypothesize that glutamate in these regions is also associated with voice intent and emotional valence of the voices. Although a previous MRS study from our laboratory did not find associations between AVH and Glx in right STG (Hjelmervik et al., 2020), this region is particularly related to processing of affective/emotional aspects of speech (Mitchell & Crow, 2005), and hence a target region to investigate with regards to emotional valence of AVH.

The current study aimed to investigate the relationship between self‐experienced and self‐reported aspects of AVHs, as measured with the BAVQ‐R questionnaire, and glutamate levels, as measured with MRS. Following previous fMRI and MRS studies of AVH frequency and severity, four brain‐regions were investigated, namely left and right STG, left IFG, and ACC (Curcic‐Blake et al., 2017; Hjelmervik et al., 2020; Hugdahl et al., 2015; see also Jardri et al., 2016 for an overview). From the studies by Strauss et al. (2018) and Sayer et al. (2000) we expected that the BAVQ‐R Malevolence, Omnipotence, and Resistance subscales (voices of negative emotional valence) and the Benevolence and Engagement subscales (positive emotional valence), would be inversely related to Glx levels, respectively. One could further hypothesize a regional dependency (Hjelmervik et al., 2020; Jardri et al., 2016), where voices of more negative emotional valence are positively related to Glx in language regions (Hjelmervik et al., 2020; Hugdahl et al., 2015); and negatively related to Glx in the ACC (Hjelmervik et al., 2020).

## 2 METHODS

### 2.1 Participants

**FIGURE 1:**
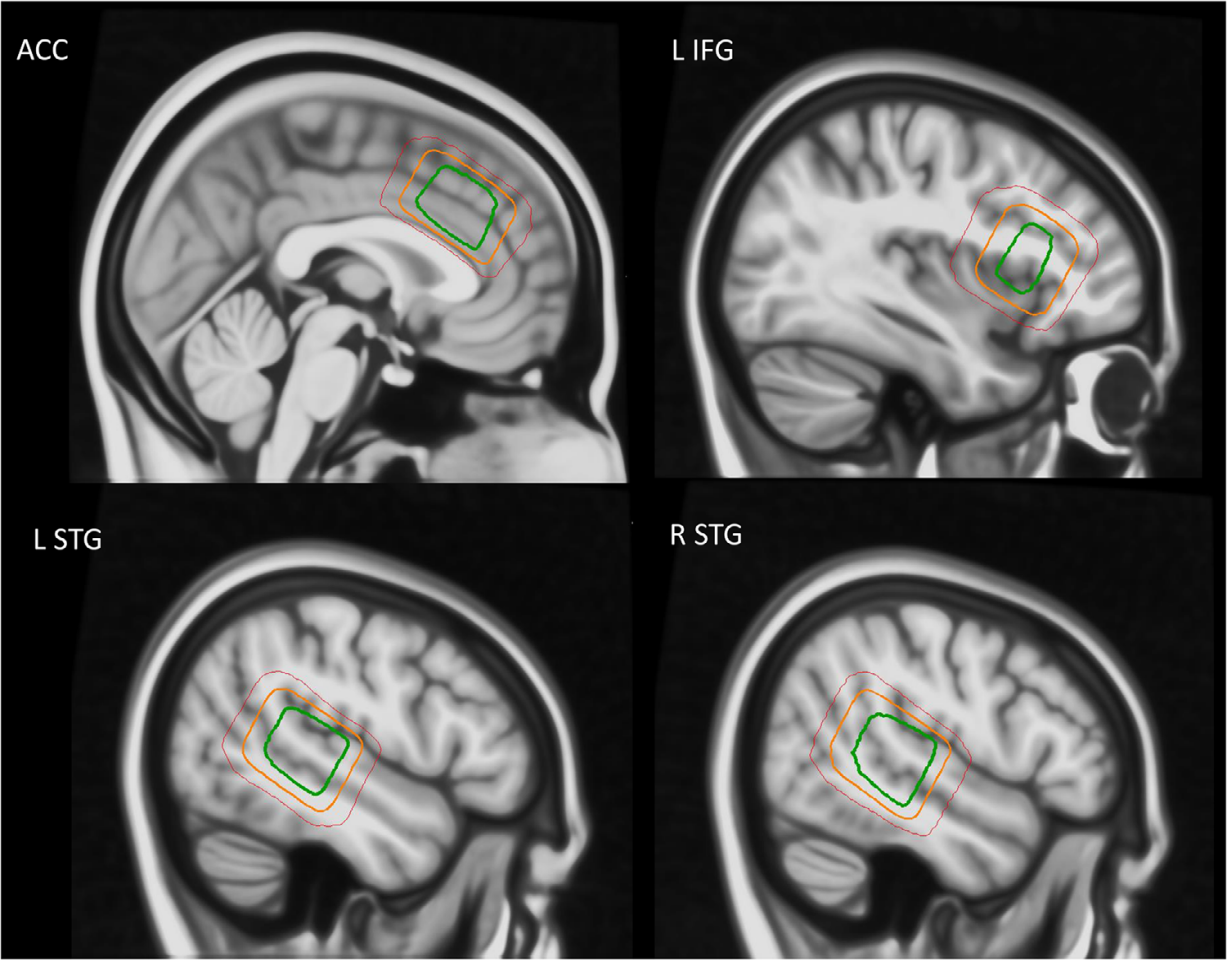
MRS measurements were conducted in four cortical regions, here displayed in sagittal view: The anterior cingulate cortex (ACC), the left inferior frontal gyrus (L IFG), the left superior temporal gyrus (L STG), and the right superior temporal gyrus (R STG). The red, orange, and green contours represent 95%, 50%, and 5% confidence regions respectively for voxel placement across the entire group

Thirty‐seven schizophrenia patients (mean age 26.84 years, SD 8.65; 11 women, and 26 men) underwent MRS scanning. The patients were recruited from the Division of Psychiatry, Haukeland University Hospital in Bergen, and surrounding local psychiatric outpatient clinics. The patients were diagnosed with schizophrenia spectrum disorder according to the ICD‐10 diagnostic manual (World Health Organization, 1992; Norwegian translation; https://ehelse.no/standarder‐kodeverk‐og‐referansekatalog/helsefaglige‐kodeverk/kodeverket‐icd‐10‐og‐icd‐11). Diagnoses were based on the structured clinical interview for DSM‐IV Axis I Disorders (SCID‐I) conducted by trained physicians/psychiatrists and psychologists. The diagnoses were converted to ICD‐10 diagnoses. All patients that were on medication used second‐generation antipsychotic medication, or second‐generation in combination with first‐generation antipsychotics (see Table 1 for more details on medication use and illness). Global severity of symptoms in the patient group as assessed by the PANSS (Kay et al., 1987) total score were 70.73, SD 14.84 (Positive‐total 18.24, SD 4.71; Negative‐total 16.49, SD 4.74; General‐total 36.00, SD 8.81). Most patients reported to have elementary school education (*n *= 21), while some had high school (*n *= 9), or college/university education (*n *= 7). To be included in the study, patients had to score 2 or higher on the PANSS P3 item. In addition, the physicians/psychiatrists performing the PANSS interview made an evaluation as to whether the patient experienced auditory hallucinations and included those who did. Two MRS data collection protocols, including varying voxel placements, were used. One protocol (hereafter referred to as Protocol 1) included MRS recordings from the left and right STG and ACC and was used in 22 patients. The next protocol (hereafter referred to as Protocol 2) included MRS recordings from the left STG and left IFG and was used in 15 patients (see Figure 1). Data from the two protocols was pooled. The sample included in the study is a subsample of patients from a larger study (Beresniewicz et al., 2021; Hjelmervik et al., 2020; Weber et al., 2021). The current sample (*n* = 37) made up ≈50% of the patients compared to the full sample (*n* = 76: unique subjects) in Hjelmervik et al. (2020). The mean PANSS P3 score for the current study sample was 3.62 (1.48 SD) in comparison to 2.64 (1.66 SD) in Hjelmervik et al. (2020). The difference in mean P3 score is mainly due to the exclusion of patients having P3 scores below 2 in the current study.

**TABLE 1:** Patient information and medication details

	*n*	M (SD)
Illness onset age	33	22.21 (5.84)
Illness duration	33	4.24 (7.36)
Smoking	21	
Medicated AP	35	DDD: 0.92 (0.68)
Medication use > 1 year	16	
Medication use < 9 mnds	9	
Medication use < 3 weeks	10	
Antidepressants	3	
Mood stabilizers	1	
Benzodiazepines	5	
Anticholinergic	1	
Unmedicated	2	

Note: The dosages  of the antipsychotics used were converted to defined daily doses, which is the assumed average maintenance dose per day for a drug used for its main indication in adults (WHOCC ‐ Definition and general considerations).

Abbreviation: AP, antipsychotic medication.

The study was approved by the Regional Committee for Medical Research Ethics at the University of Bergen (REK no 2016/800) and conducted according to the Declaration of Helsinki. All participants received oral and written information about the study before signing a written consent form.

### 2.2 Beliefs about voices questionnaire (BAVQ‐R)

The BAVQ‐R questionnaire (Chadwick et al., 2000) was administered to the patients prior to MRS scans. This questionnaire consists of 35 items that are rated on a four‐point scale from “disagree” ( = 0) to “strongly agree” ( = 3). There are six statements for each of the three sub‐scales that are related to beliefs about voices; Malevolence scale (e.g., “My voice wants to harm me”); Benevolence scale (e.g., “My voice wants to protect me”); and Omnipotence scale (e.g., “My voice is very powerful”). In addition, the questionnaire includes nine and eight statements, respectively, for the Resistance and Engagement sub‐scales, which concerns how the patients relate to and cope with their voices. Each of these two sub‐scales is further subdivided into an emotional component (Emotional Resistance e.g., “My voice frightens me”; Emotional Engagement e.g., “My voice reassures me”) and a behavioral component (Behavioral Resistance e.g., “When I hear my voice, usually I tell it to leave me alone”; Behavioral Engagement e.g., “When I hear my voice, usually I listen to it because I want to”). Patients that experience more than one voice, are instructed to complete the questionnaire for their “dominant voice.”

### 2.3 MR Image acquisition

A 3T GE‐Signa MRI scanner was used for data collection. First, an anatomical T1‐weighted image was acquired of each subject (3D Enhanced Fast Gradient Echo (EFGE3D sequence). For Protocol 1, the following parameters were used: Repetition time (TR)/echo time (TE)/flip angle (FA)/field of view (FOV) 7.8 ms/2.9 ms/14°/256 mm, 256 × 256 scan matrix, 188 sagittal slices, voxel size 1 × 1 × 1 mm). For Protocol 2, a TR of 3.0, TE of 6.8, and FA of 12 was used, otherwise parameters were the same as for Protocol 1. Thereafter, MRS acquisition was performed. In Protocol 1, short echo time ^1^H‐spectra were obtained from the left and the right STG (voxel size 24 × 40 × 30 mm) and ACC (voxel size 40 × 40 ×25 mm) by using a single‐voxel point‐resolved spectroscopy (PRESS) sequence (TE/TR = 35 ms/1500, 128 repetitions). Protocol 2 contained acquisitions from the left STG (voxel size 24 × 30 ×31 mm) and left IFG (24 × 38 × 28 mm) obtained by using a PRESS sequence with identical parameters as for Protocol 1 except for left STG voxel‐size. Unsuppressed water reference spectra (eight repetitions) were acquired automatically after the acquisition of water‐suppressed metabolite spectra in both protocols. Center‐of‐mass for the voxel localization, given in Montreal neurological Institute (MNI) space *x*, *y*, *z* coordinates was for the ACC: 0.389, 25.4, 33.9 mm, left IFG: −36.8, 18.9, 11.9 mm, left STG: −48.3, −35.9, 6.02 mm, and for the right STG: 50.2, −34.2, 6.11 mm. Between Protocol 1 and 2, a scanner upgrade was conducted that included a change of head‐coil from 8 to 32 channels.

### 2.4 Data analysis

MRS data were analyzed using the LCModel version 6.3‐1J (Provencher, 1993) software. A standard basis‐set was used, including components from 15 metabolites (Alanine, Aspartate, Creatine, γ‐aminobutyric acid (GABA), Glucose, Glutamine, Glutamate, Glycerophosphorylcholine, Phosphorylcholine, Lactate, myo‐inositol, N‐acetylaspartate, N‐acetyl‐aspartate‐glutamate, scyllo‐inositol, and Taurine. Default baseline parameters and a default set of simulated macromolecule components were included to model background signal underlying the metabolite spectrum. The estimates of the metabolites were thereafter scaled to an internal water reference. Adjustments were made to account for differing water concentration in the different tissue classes, partial volume effects, metabolite relaxation times and differing water relaxation times between the tissue classes, using the formula of (Gasparovic et al., 2006). The segmentation tool of the Statistical Parametric Mapping (SPM8) software (www.fil.ion.ucl.ac.uk/spm) was used to extract information on tissue content within the spectroscopy voxel on the basis of the T1 image. A local quality control procedure identified three spectra (two from the right STG and one from ACC) that were excluded from further analysis. Multiple factors were considered in the assessment of spectra quality: Signal‐to‐noise ratio (SNR), spectral linewidth (FWHM; see Table 2), and CRLB %SD of estimates for key metabolites, in addition to assessment of variance and magnitude of features in the residuals after fitting, and magnitude of aberrant features in the spectrum (relative to group mean spectrum). The resulting quality score flagged spectra of concern, that were subject to further visual scrutiny of the fit and residuals, to identify spectra which were of insufficient quality for meaningful assessment. For further details on analysis procedure, see Hjelmervik et al. (Hjelmervik et al., 2020; 2018). In the current study the combined measure of glutamate and glutamine (Glx) levels was reported (see Table 2).

**TABLE 2:** Concentration means and standard deviations of Glx, glutamate, glutamine (institutional units), and data quality parameters in the four cortical regions

		N	Mean	SD
Left STG	Glutamate	34	13.43	2.01
	Glutamine	34	3.97	2.81
	**Glx**	**37**	**16.52**	**3.88**
	Glx CRLB	37	7.27	1.82
	SNR	37	37.95	12.69
	FWHM (Hz)	37	8.57	2.56
Left IFG	Glutamate	15	14.52	1.90
	Glutamine	15	3.19	2.24
	**Glx**	**15**	**17.33**	**3.66**
	Glx CRLB	15	6.60	1.45
	SNR	15	46.33	8.28
	FWHM (Hz)	15	7.06	1.57
ACC	Glutamate	19	16.65	1.80
	Glutamine	19	3.79	2.07
	**Glx**	**22**	**19.58**	**2.88**
	Glx CRLB	22	6.32	1.13
	SNR	22	41.64	6.30
	FWHM (Hz)	22	8.03	1.95
Right STG	Glutamate	18	16.16	1.91
	Glutamine	18	7.38	4.26
	**Glx**	**21**	**21.55**	**4.50**
	Glx CRLB	21	6.14	1.56
	SNR	21	33.57	9.70
	FWHM (Hz)	21	9.32	2.59

Abbreviations: ACC, anterior cingulate cortex; L IFG, left inferior frontal gyrus; L STG, left superior temporal gyrus; R STG, right superior temporal gyrus; SNR, signal‐to‐noise ratio; FWHM, full‐width at half maximum (linewidth). There is a difference in *N* between Glx and glutamate/glutamine due to cases where the composite signal was inseparable.

### 2.5 Statistical analysis

Glx values were subjected to statistical analysis using Linear mixed models available in the SPSS software package (https://www.ibm.com/analytics/spss‐statistics‐software). Five multivariate models were applied to the data in order to test the linear relationship between regional Glx and emotional valence of AVHs. In these models, Glx served as dependent variable, brain Region was entered as a repeated fixed factor, and the BAVQ‐R sub‐variables Malevolence, Benevolence, Omnipotence, Resistance, Engagement) were entered as regressor variables (one for each model). FDR correction (Benjamini & Hochberg, 1995) was done to control for multiple comparisons (https://www.webcitation.org/5s004b7CI (webcitation.org); Pike, 2011), and adjusted *p*‐values are reported in addition to the uncorrected *p*‐values. In order to control for the scanner up‐grade, this was included as a covariate dummy variable in each of the analyses. In addition, participants age was implemented as a covariate (Marsman et al., 2013). Effect sizes are reported as unstandardized beta values.

## 3 RESULTS

**FIGURE 2:**
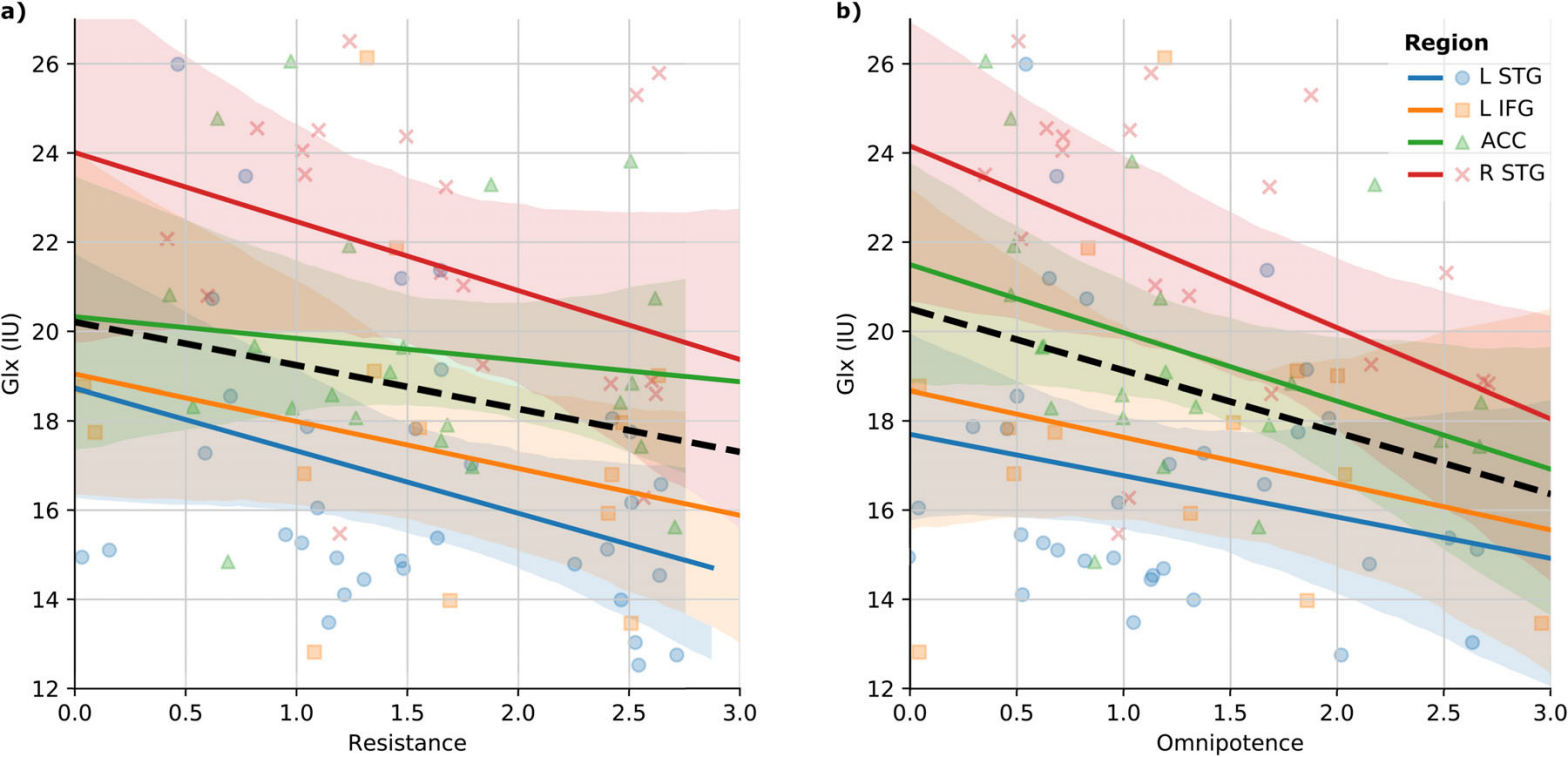
Graphs illustrating relations between Glx (*y*‐axis) and the BAVQ‐R sub‐variables (*x*‐axis) omnipotence, and resistance across the four cortical regions ACC, left IFG, left STG, and right STG. Note that relationships between variables are illustrated using fixed predicted values from the LMMs (*y*‐axis). Abbreviations: ACC, anterior cingulate cortex; IU, institutional units; L IFG, left inferior frontal gyrus; L STG, left superior temporal gyrus; R STG, right superior temporal gyurs;

Multivariate analyses were conducted for each BAVQ‐R sub‐scale (see Figure 2).

In the first analysis, a significant main‐effect of Omnipotence (*F*(1,53.39) = 7.88, *p* = .01, FDRcorr *p* = 0.04, *B* = −1.92) was found, reflecting a negative association between Omnipotence and Glx across brain regions. Further, there was a significant main‐effect for the sub‐scale Resistance (*F*(1,53.13) = 6.02, *p* = .02, FDRcorr *p* = 0.04, B = −1.33), suggesting a significantly negative association between Resistance and Glx levels across regions. As the Resistance sub‐scale consists of both a behavioral component (questions addressing responses to the perceived voice intentions) and an emotional component (questions addressing emotional responses), post‐hoc analyses were conducted by splitting the Resistance factor. Only the emotional component (F(1,52.85) = 7.06, *p* = .01, FDRcorr *p* = 0.02, B = −.67) was found significantly associated with Glx. There were no significant effects (after FDR correction) for the sub‐scales Malevolence (*F*(1,44.77) = 4.33, *p* = .04, FDRcorr *p* = 0,07, B = −.89), Benevolence (F(1,31.46) = .85, *p* = .36, FDRcorr *p* = 0.45, B = −1.07) and Engagement (F(1,32.42) = .15, *p *= .70, FDRcorr *p =* 0.70, B = −.09). For the latter analysis a main‐effect of Region (F(3,46.36) = 2.85, *p *= 0.05) was found, reflecting higher levels in the right as compared to the left STG. Moreover, no further main effects of Region or interaction effects between Region and the BAVQ‐R sub‐scales were found significant for any of the analyses. The covariate variables controlling for the change of coil (all *p* < 0.02) and Age (all *p* < 01) were found significant in all analyses.

## 4 DISCUSSION

The current study investigated self‐reported emotional valence of AVHs in relation to glutamate levels (measured as Glx) in temporal and frontal brain regions. The results showed that voices experienced to be omnipotent, and which the patients resisted, were negatively associated with Glx levels. This was found across all four brain regions (see Figure 2). Further exploration of the resistance factor with post hoc analyses showed that it was the emotional component of the resistance sub‐scale (e.g., feeling of anxiety or fear in relation to the voice) that was driving the significant effect.

The expected findings of the study were partly confirmed. Indeed, significant relationships were found between Glx and the negative emotional valence sub‐scales omnipotence and emotional resistance. However, the prediction of a reversed relationship between Glx and benevolence and engagement did not come through. This suggests that it is the negative emotional valence of AVH that is of essence, where those patients who experienced voices that were omnipotent and that evoke emotional resistance showed reduced Glx levels across frontal and temporal brain regions. While previous studies have identified a relationship between Glx and severity of AVHs (e.g., Hjelmervik et al., 2020; Hugdahl et al., 2015), the current study adds to this knowledge by suggesting an association between Glx and negative emotional valence of the voices, as experienced and reported by the patient. The fact that all three negative valence sub‐scales, Omnipotence, Resistance, and Malevolence (not significant after correction), were negatively associated with Glx, suggests high internal consistency in the data and strengthens the credibility of the results.

The hypothesis that the relationship between emotional valence of AVH and Glx would be dependent on region, was not confirmed. Instead, reduced Glx levels in relation to negative emotional valence were found across all four regions. Previous findings have shown reduced Glx in the ACC in patients with increasing AVH severity (Hjelmervik et al., 2020). The current finding that reduced ACC Glx is associated with increased negative emotional valence would support this finding. However, the current finding of reduced Glx with more negative emotional valence also in the left STG, could be argued to stand in contrast to previous findings that suggest an increase in left STG Glx with increasing AVH severity (Hjelmervik et al., 2020; Hugdahl et al., 2015). The difference in AVH measurement (PANSS P3 vs. BAVQ‐R) could explain this discrepancy. Hjelmervik et al. (2020) suggested that patients had generally reduced STG “baseline” glutamate levels relative to controls (Bustillo et al., 2020), which again, for hallucinating patients, rose to normal levels by the neuronal activity associated with the AVHs (Hjelmervik et al., 2020). Characteristics picked up by the P3 scale, such as frequencies and loudness of voices, might be driving this neuronal activity (and thereby the Glx levels) to a larger extent than the emotional valence in the voices. It has for example been shown in other studies that emotionally negative content of real voices does not cause higher neuronal activity in auditory cortical regions than positive content (Bestelmeyer et al., 2017). It could also be that BAVQ picks up on a potentially underlying phenomenon such as the emotional state of the patient, for example, depression or anxiety (Chadwick et al., 2000; Mawson et al., 2010), which again relates to reduced Glx. In this connection it is interesting to mention that reduced Glx has been found in depression using meta‐analytical approaches (Luykx et al., 2012). In trait depression Glx levels in ACC was found reduced, but also during depressive episodes where reduced Glx was found in multiple brain regions. This suggests that state‐dependent fluctuations in glutamate signaling may be present during depression. Finally, it should be considered that sample differences in the two studies could explain the differences in findings, that is, that the current sub‐sample might not be representative for the full sample in Hjelmervik et al. (2020). For example, in the current study only hallucinating patients (PANSS P3 = / > 2) were included, whereas Hjelmervik et al. (2020) included a significant portion of nonhallucinating patients. Removing patients at the extreme end of the scale (P3 scores of 1) could affect the regression line.

What underlying cellular mechanism may be related to reduced Glx‐levels in association with negative emotional valence can at present only be a matter of speculation. Metabolic cycles of glutamate (Bak et al., 2006; Gaisler‐Salomon et al., 2009) could be affected, or as commonly suggested, reduced glutamate could be a consequence of NMDA‐receptor hypofunction (Marsman et al., 2013). The latter explanation is in line with findings of induced schizophrenia‐like symptoms from ketamine and other NMDA‐receptor antagonists (Adler et al., 1999; Krystal et al., 1994; Lahti et al., 2001). Similarly, the current results could be interpreted in terms of more pronounced NMDA hypofunction in the patients experiencing emotionally negative voices, resulting in generally reduced inter‐neuronal signal transmission. As a result of NMDA hypofunction, Glx levels might be downregulated through a negative‐feedback mechanism, or through an excitatory/inhibitory imbalance which in the long run can lead to neurotoxicity, gray matter atrophy and reduced Glx levels (Plitman et al., 2014).

The reduced Glx levels as a function of negative emotional valence in AVH appear to be a global rather than a region‐specific phenomenon. This indicate that the findings point to a more basic/underlying neuropathology of the disease (as discussed above) related to negative emotional valence. This could involve hypofunction of prefrontal cortical regions, in particular the ACC (Minzenberg et al., 2009) accompanied with reduced executive functions (Carter et al., 1996; Hugdahl et al., 2013) Impaired executive control has repeatedly been demonstrated in schizophrenia, and has been suggested to result in failure to suppress AVHs arising bottom‐up from language regions (Hugdahl, 2009). Similarly, in the current study, reduced ACC Glx could cause neuronal hypoactivity and a failure to cognitively control AVH. In this sense, the patients’ experiences of voice omnipotence and emotional resistance could be a reflection of the loss of control. It should also be noted that ACC is involved in emotion regulation through inhibitory signals to the amygdala (Jhang et al., 2018). Future studies aiming to address the relationship between negative emotional valence of voices and brain chemistry should therefore attempt to measure Glx also in the amygdala. The finding that emotional valence of AVHs predicts Glx‐levels, and hence glutamate levels, could be relevant for the idea of deep phenotyping of patients’ symptoms as suggested by Sommer et al. (2018). According to this idea, it would be of essence to identify symptoms that could act as predictors for underlying psychopathology which, down the road, could be informative in tailoring individual treatment.

## 5 LIMITATIONS

A few limitations of the study need to be addressed. First, a change of head‐coil was implemented during data collection resulting in data being collected with two different coils. The change of head‐coil might have altered the accuracy in measurement of Glx: SNR and linewidth were somewhat improved after this change. It is, however, unlikely that such a change would impact the relationship between Glx and negative valence of AVH that was observed in the study. Nevertheless, the inclusion of coil as a covariate should have controlled for any systematic effect on Glx levels. Second, additional variables could potentially have influenced the results by modifying Glx levels (Merritt et al., 2021), including medication variables such as medication type(s), dosage, response, duration/medication onset. We cannot exclude that such variables have impacted the results, however, a quick analysis of medication dosage against regional Glx levels suggested no effect (*p* = 0.86). Third, the current study chose to report Glx, as the combined signal of glutamate and glutamine—this could be assessed with more certainty than the discrete components, therefore allowing us to include more patients. This comes with the uncertainty as to whether the results are driven by changes in Glutamate or in glutamine, or both.

## 6 CONCLUSION

Negative emotional valence of hallucinatory voices has previously been shown to be an important predictor of illness severity and need for care in schizophrenia. The current study investigated a potential role of glutamate in negative emotional valence of AVH. The results suggest that voices that are believed by the patients to be omnipotent, and which the patients attempt to emotionally resist, are negatively related to Glx levels in temporal and frontal brain regions. This might indicate that high negative emotional valence in voices, as experienced by schizophrenia patients, is related to an underlying dysfunction in the glutamatergic system.

## FUNDING SOURCE

In the current study, the contributions of coauthors H.H., ARC, JJB, and K.H. was funded with a grant from ERC #249516. In addition, the study was funded by grants from the Norwegian Research Council (RCN) grant nr. 13727 and the Western Norway Regional Health Authority (Helse‐Vest Samarbeidsorganet) grants nr. 911820 and 911629 to E.J.

## CONFLICT OF INTERESTS

The coauthors A.R.C., L.E., R.G., and K.H. own shares in the NordicNeuroLab Inc. company, which produces some of the add‐on equipment used during data acquisition. A.R.C., L.E., R.G., and K.H., as all other authors, declare no conflict of interest.

### PEER REVIEW

The peer review history for this article is available at https://publons.com/publon/10.1002/brb3.2446

## Data Availability

According to Norwegian law, data sharing requires approvals from the Regional Committees for Medical and Health Research Ethics, and from the Data Protection Officer at Haukeland University Hospital, on the basis of specific research proposals.
